# Unraveling the link between early sexual initiation and endometriosis: evidence from population-based analyses and genetic causal inference

**DOI:** 10.1186/s12958-026-01539-8

**Published:** 2026-03-03

**Authors:** Mengying Li, Yimei Ma

**Affiliations:** 1 Hannover Medical School, Gynecology Research Unit, Carl-Neuberg-Str.1, Hannover, 30625 Germany; 2 West China Second University Hospital of Sichuan University, Key Laboratory of Birth Defects and Related Diseases of Women and Children (Sichuan University), Ministry of Education, Chengdu, People’s Republic of China; 3 Department of Hematology and Oncology, West China Second University Hospital of Sichuan University, Chengdu, People’s Republic of China

**Keywords:** AFS endometriosis, Population-based study, Genetic epidemiology

## Abstract

**Background:**

This study investigates whether the timing of first sexual intercourse is associated with the risk of developing endometriosis and adenomyosis, aiming to shed light on early-life reproductive factors that may influence women's long-term gynecological health.

**Materials and methods:**

We analyzed data from two large population-based cohorts, the National Health and Nutrition Examination Survey (NHANES) and UK Biobank using multivariable logistic regression. In addition, we performed a two-sample Mendelian randomization analysis based on GWAS summary statistics for age at first sexual intercourse (AFS) from Mills et al. and for endometriosis-related outcomes from the FinnGen consortium, to evaluate the relationship between AFS and endometriosis. Further, genetic instruments were applied to assess the potential relationship between AFS and endometriosis, including specific subtypes such as ovarian endometriosis and peritoneal endometriosis, and adenomyosis. The primary analysis method employed was the inverse-variance-weighted (IVW) approach. To ensure robustness, the study accounted for potential horizontal pleiotropy and conducted heterogeneity tests.

**Results:**

The multivariable logistic regression analysis revealed that in the NHANES cohort, women who had their first sexual intercourse at late adolescence had a significantly higher risk of endometriosis (adjusted model, odds ratio [OR] = 1.31, 95% confidence interval [CI]: 1.01–1.70, *P* = 0.04) compared to those who initiated sexual activity in adulthood. In the UK Biobank cohort, the association was stronger: late adolescent AFS was associated with a 51% higher risk (adjusted model, OR = 1.51, 95% CI: 1.41–1.61, *P* < 0.001), and early adolescent AFS with a 73% higher risk (adjusted model, OR = 1.73, 95% CI: 1.48–2.02, *P* < 0.001). The IVW Mendelian randomization analysis demonstrated that delayed AFS was significantly associated with an about 30% lower risk of endometriosis (OR = 0.70, 95% CI: 0.57–0.86, *P* = 6.08 × 10^–4^) and with an about 48% lower risk of adenomyosis (OR = 0.52, 95% CI: 0.37–0.73, *P* = 1.37 × 10^–4^). Our study yielded no evidence of horizontal pleiotropy or heterogeneity.

**Conclusions:**

Early sexual initiation markedly increases the risks of developing endometriosis and adenomyosis, while delayed initiation may offer protective benefits. These findings highlight the potential long-term reproductive health implications of adolescent sexual behavior and support the value of preventive strategies and early education.

**Supplementary Information:**

The online version contains supplementary material available at 10.1186/s12958-026-01539-8.

## Background

Endometriosis is a global burden disease, which impacts ~ 10% (190 million) of women of reproductive age worldwide [1, 2]. This oestrogen-dependent chronic disease is linked to severe pelvic pain, abdominal bloating, nausea, fatigue, and can sometimes lead to depression, anxiety, and infertility [3–7]. Pathologically, it is characterized by the presence of endometrial-like tissue outside the uterine cavity, most commonly involving the ovaries and pelvic peritoneum. Adenomyosis, a related but distinct condition, is defined by the presence of endometrial glands and stroma within the myometrium, leading to uterine enlargement, abnormal uterine bleeding, and dysmenorrhea [8]. Although endometriosis and adenomyosis have long been considered distinct diseases, growing studies indicate that they share similar clinical features and risk factors, and often occur together in the same patients. Both conditions contribute to reduced quality of life and significant economic burden [9–14]. Despite their prevalence and clinical importance, the common etiological factors or triggers remain unclear, resulting in limited therapeutic option [15–17].

Importantly, while the clinical diagnosis of endometriosis is often delayed by 8–10 years after symptom onset, a growing body of evidence indicates that disease initiation frequently occurs during adolescence or early adulthood [18–20]. Adolescent endometriosis has been increasingly recognized, with laparoscopically confirmed disease reported in adolescents presenting with severe dysmenorrhea and chronic pelvic pain [10, 21]. Similarly, although adenomyosis has historically been considered a disease of multiparous or older women, advances in imaging techniques including high-resolution transvaginal ultrasound have revealed that adenomyosis-like features can be detected in younger women, including adolescents and young adults [9, 19]. These observations suggest that early-life and adolescent exposures may play a critical role in the initiation and progression of both conditions. Recent studies have started to uncover the complex interplay between early sexual activity and the development of these gynecological conditions [22–26]. Early sexual activity may expose individuals to biological and environmental factors, such as hormonal fluctuations, infections, and physical trauma during adolescence, that adversely influence the onset of reproductive health conditions [27]. Sexually active women tend to have elevated concentrations of estrogen and progesterone [28]. Estrogen, especially 17β-Estradiol (E_2_), promotes the growth and persistence of endometriotic tissue and is key to the associated inflammation and pain in endometriosis [3]. The disease is also characterized by altered expression of steroid receptors, including increased estrogen receptor β (ERβ) in lesions, and a noted progesterone resistance in stromal cells, which compromises cellular interactions and responses [29]. The psychological stress associated with early sexual activity, particularly if it is non-consensual or occurs in an unsupported environment, can affect the hypothalamic-pituitary–gonadal (HPG) axis and lead to hormonal dysregulation [30]. Current studies indicate an association between the AFS and the risk of developing endometriosis [31]. However, the causal relationship remains unexplored.

Understanding this relationship could lead to better prevention strategies, earlier diagnosis, and more effective treatments for those affected. To investigate the association between early sexual behavior and endometriosis, we performed a logistic regression analysis using data from the National Health and Nutrition Examination Survey (NHANES) and UK Biobank cohorts [32]. In addition, two-sample Mendelian randomization analyses were conducted using GWAS summary statistics for AFS and endometriosis-related outcomes to verify the causal relationship.

## Materials and methods

### Overall study theoretical framework

**Fig. 1:**
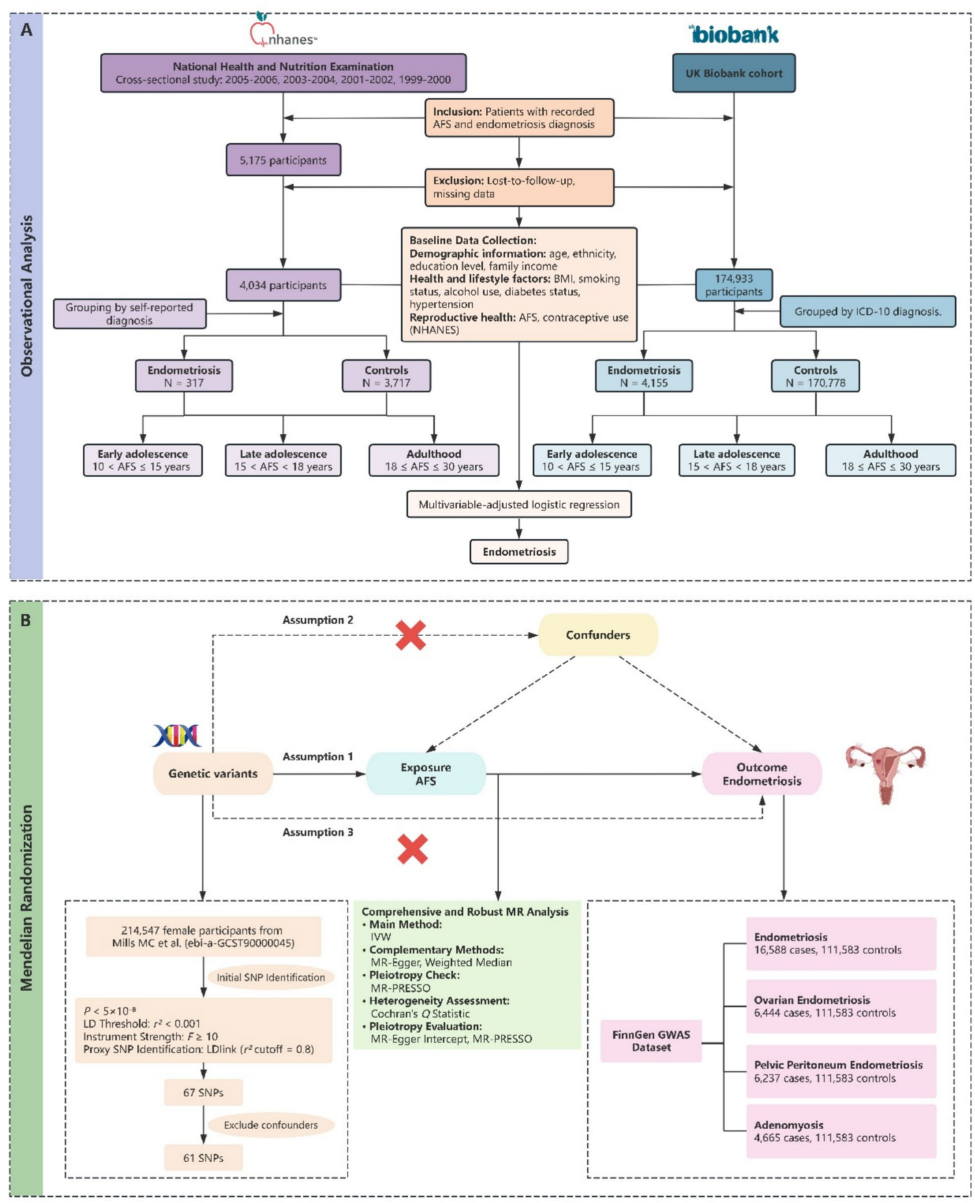
The detailed process of the study. **A** Observational analysis using the NHANES and the UK Biobank cohorts to examine the association between AFS and endometriosis. **B** MR analysis assessing the causal effect of AFS on endometriosis. NHANES: National Health and Nutrition Examination Survey; AFS: Age at first sexual intercourse; BMI: Body mass index; SNP: Single nucleotide polymorphisms; LD: Linkage disequilibrium; IVW: Inverse variance-weighted; GWAS: Genome-wide association studies

The present study was conducted in two stages, as illustrated in Fig. 1. In the first stage (panel A), two population-based cohorts were analyzed: the NHANES (United States) and the UK Biobank (United Kingdom). NHANES is a continuous, nationally representative survey of the non-institutionalized U.S. population that integrates standardized interviews, physical examinations, and laboratory measurements, with public-use data released in two-year cycles, whereas the UK Biobank is a large prospective cohort of approximately 500,000 UK participants with extensive baseline phenotyping and genotyping, and longitudinal outcome ascertainment through linkage to routinely collected health records [33, 34]. A multivariable regression analysis was performed to examine the association of AFS with endometriosis. In panel B, we assessed the causal effect of genetically determined AFS on endometriosis using MR analysis of summary statistics data from genome-wide association studies (GWAS).

### Study population for observational analyses

Eligible participants were women with available data on AFS and endometriosis diagnosis. Participants were included if they had complete information on the exposure, outcome, and relevant covariates. Individuals with missing data on AFS or endometriosis diagnosis were excluded.

The exposure of interest was AFS, categorized into early adolescence (10 < AFS ≤ 15 years), late adolescence (15 < AFS < 18 years), and adulthood (18 ≤ AFS ≤ 30 years). The primary outcome was physician-diagnosed endometriosis, ascertained through self-report in the NHANES and hospital records in the UK Biobank. Adenomyosis was evaluated as an outcome in the MR analysis using GWAS summary statistics. Key covariates included demographic, socioeconomic, lifestyle, and health-related factors, as described below.

This study utilized data from two large-scale population-based cohorts: the NHANES and the UK Biobank. For the NHANES component, we conducted a cross-sectional study utilizing data from NHANES spanning four cycles: 1999–2000, 2001–2002, 2003–2004, and 2005–2006. Data for AFS below 10 years old are extremely rare and may be associated with special circumstances (e.g., sexual abuse), while data for AFS above 30 years old are considered outliers due to their low frequency, potentially leading to unstable statistical estimates. Including extreme values (AFS < 10 or > 30) could introduce outliers or bias, adversely affecting the stability and reliability of the regression model. Limiting the age range helps minimize these issues, resulting in more representative and robust analytical outcomes. Therefore, we limited our study to participants whose AFS was between 10 and 30 years old because most individuals initiate sexual activity during this period.

We extracted questionnaire-based information from the NHANES database, including demographic information (ethnic and family income), health and lifestyle factors (body mass index (BMI), smoking status, alcohol use, hypertension and diabetes status), reproductive health (AFS, contraceptive use such as used DepoProvera or injectables). A non-smoker was defined as an individual who has smoked fewer than 100 cigarettes over their lifetime. Those who have smoked more than 100 cigarettes, but do not currently smoke were classified as former smokers, while those who currently smoke regularly were classified as current smokers. In terms of alcohol consumption, participants were categorized as non-drinkers (0 drinks per year), moderate drinkers (less than 1 drink per day), and heavy drinkers (1 or more drinks per day) [35]. These participants were diagnosed into two groups: endometriosis group and control group.

The diagnosis of endometriosis in NHANES participants was determined using mobile examination center (MEC) information on reproductive health. Female participants between 20–54 years old were asked “Has a doctor or other health professional ever told you that you had endometriosis? (Endometriosis is a disease in which the tissue that forms the lining of the uterus/womb attaches to other places, such as the ovaries, fallopian tubes, or abdominal cavity”), of which who reported “Yes” were classified as having endometriosis (details in https://www.cdc.gov/nchs/nhanes/index.htm). According to the information of the questionnaire, a binary variable (no, yes) for endometriosis diagnosis was established. Among 5175 eligible female participants from the NHANES cycles, individuals with missing data on AFS, endometriosis diagnosis, or key covariates were excluded, resulting in a final analytical sample of 4,034 participants.

For the UK Biobank component, we included data from 174,933 women who had available information on AFS and endometriosis diagnosis. UK Biobank baseline data were collected between 2006 and 2010. AFS and covariate data were collected via touchscreen questionnaires at baseline assessment centers across the United Kingdom. Participants were similarly categorized into the three AFS groups (early adolescence, late adolescence, and adulthood). Diagnosis of endometriosis was identified from linked hospital records using the International Classification of Diseases 10th Revision (ICD-10) code. Covariates included ethnicity, household income, BMI, education level, smoking and alcohol consumption, Townsend deprivation index, hypertension, and type 2 diabetes status. After data cleaning and exclusion of incomplete records, 4,155 women with endometriosis and 170,778 controls were included in the analysis.

### Observational analysis based on cohort data

Descriptive statistics were employed to summarize the demographic and clinical characteristics of the study population. Chi-square tests were utilized to examine the relationship between endometriosis and categorical variables. Continuous variables were assessed for distributional characteristics; variables with approximately normal distributions are presented as mean ± standard deviation (SD) and compared using Student’s t-test, whereas non-normally distributed variables are presented as median [interquartile range (IQR)] and compared using the Mann–Whitney U test. Multivariable logistic regression models were used to investigate the association between AFS and the risk of developing endometriosis. Model 1 was an unadjusted model including AFS only and was used to estimate the crude association. Model 2 was adjusted for a prespecified set of covariates selected based on prior knowledge and potential confounding relationships, rather than solely on statistical significance in baseline comparisons. For NHANES, model 2 included ethnic, annual family income, BMI, smoking status, alcohol consumption, hypertension, diabetes and used Depo-Provera or injectables. For UK Biobank data, model 2 was adjusted for ethnic, education, income, BMI, smoking status, alcohol consumption, Townsend deprivation index, hypertension and type 2 diabetes mellitus. Diabetes was included in both cohorts as a marker of metabolic health and healthcare contact, which may confound associations between early-life exposures and later gynecological disease risk. The likelihood ratio test (LRT) was used to compare two nested models in NHANES—unadjusted model 1 and a more complex model 2 that included additional predictors. The LRT assessed whether the extra covariates in model 2 significantly improved the model's fit to the data. Given the very large sample size of the UK Biobank, formal LRT comparisons were not emphasized, as even trivial differences are expected to yield statistically significant results; therefore, we focused on adjusted effect estimates for interpretability. All analyses were conducted using R (version 4.1.3; Lucent Technologies; New Jersey, USA). Significance levels were calculated using two-sided tests, with a threshold for significance set at *P* < 0.05. No formal a priori sample size calculation was performed for the observational analyses, as all eligible participants from NHANES and the UK Biobank were included, and the large sample sizes provided sufficient statistical power to detect clinically meaningful associations.

### AFS as exposure in MR analysis

Data from the largest published GWAS datasets were used, encompassing AFS in 214,547 female participants from the UK Biobank (Mills MC et al., study ebi-a-GCST90000045) [36]. Since endometriosis is a gynecological condition, we focused exclusively on female participants. SNPs were selected based on a stringent genome-wide significance level (*P* < 5 × 10^–8^) and were employed as instrumental variables (IVs). To mitigate the impact of strong linkage disequilibrium (LD), we employed a stringent LD threshold (*r*^2^ < 0.001). The strength of these IVs was evaluated using the *F* statistic, and any SNP with *F* < 10 was excluded due to weak instrument strength. For SNPs not present in the GWAS dataset, the LDlink platform was used to identify suitable proxy SNPs, applying a common LD *r*^2^ cutoff of 0.8. This approach ensures that the genetic instruments used in this analysis are both comprehensive and robust. Additionally, we utilized the PhenoScanner database (http://www.phenoscanner.medschl.cam.ac.uk/) to meticulously exclude 6 SNPs that are specifically associated with outcomes such as endometriosis and potential confounders like BMI. PhenoScanner is a comprehensive resource for exploring associations between genetic variants and phenotypes, helping to ensure that our analysis remains robust and unbiased by these confounding factors. This selection criteria resulted in identifying 61 SNPs that were significantly associated with AFS, which we included in our analysis. Details of these SNPs can be found in Supplementary Table 1.

### Endometriosis and adenomyosis as outcomes in MR analysis

Furthermore, the GWAS of endometriosis was obtained from the release 10 of the FinnGen biobank. The FinnGen Consortium aims to collect and analyze genome and health data from 500,000 Finnish biobank participants, integrating nationwide Finnish health registry data with diagnoses recorded from 1998 onwards. Cases were identified based on preliminary diagnoses using ICD-10 diagnostic codes. Endometriosis cases were identified using ICD-10 diagnostic codes N80, and adenomyosis was identified using the ICD-10 code N80.0 (endometriosis of the uterus), reflecting clinical diagnoses based on imaging techniques like transvaginal ultrasound or magnetic resonance imaging (MRI), surgical and histopathological confirmation. Controls were defined as individuals without recorded diagnoses of endometriosis or adenomyosis. Specifically, the endometriosis group included 16,588 cases and 111,583 controls, while the adenomyosis (endometriosis of the uterus) group comprised 4,665 cases and 111,583 controls. Additionally, the endometriosis of the ovary subgroup consisted of 6,444 cases and 111,583 controls, endometriosis of the pelvic peritoneum included 6,237 cases and 111,583 controls (Supplementary Table 2). More detailed information on this GWAS is available on the FinnGen website (https://www.finngen.fi/).

### MR methods and sensitivity analysis

Formal a priori sample size calculations were not conducted, as the analyses were based on fixed sample sizes from large population-based cohorts and publicly available GWAS summary statistics. For MR analysis, the inverse-variance weighted (IVW) method was primarily employed due to its efficiency in providing a comprehensive estimate of the causal effect by combining information across multiple genetic variants. We also utilized complementary methods such as MR-Egger and the weighted median, which offers a robust estimate of the causal effect.

To further ensure the robustness of our findings, we used MR-PRESSO test to identify and correct for horizontal pleiotropy by detecting outlier SNPs that contribute to pleiotropic effects, thereby providing a corrected causal estimate. Heterogeneity among the genetic instruments was assessed using Cochran's *Q* statistic, and pleiotropy was evaluated through the MR-Egger intercept test and the MR-PRESSO global test. These methods collectively ensure that our analysis is comprehensive and robust, accounting for potential pleiotropy and heterogeneity to provide more reliable results and valid conclusions.

## Results

### Population characteristics of NHANES

This study included 4,034 participants from the NHANES, comparing women diagnosed with endometriosis (*N* = 317) to a control group (*N* = 3,717). AFS is presented here to illustrate its distribution by outcome status; however, it was treated as the exposure variable of interest in all analytical models rather than as a descriptive baseline characteristic. Participants were categorized into three groups based on their AFS: early adolescence (10 < AFS ≤ 15 years old, 11.4% in the endometriosis group vs. 14.4% in controls), late adolescence (15 < AFS < 18 years old, 51.7% vs. 44.2%) and adulthood (18 ≤ AFS ≤ 30 years old, 36.9% vs. 41.4%). There were no significant differences between the two groups in terms of annual family income, BMI, alcohol consumption and diabetes status. There was a significant difference in smoke status, hypertension and DepoProvera or injectables between two groups. The detailed results of the NHANES population characteristics are shown in Table 1.

**Table 1:** Baseline characteristics of included patients from NHANES

	Endometriosis (*N* = 317)	Controls (*N* = 3,717)	*P* value
AFS group^*^, n (%)			0.029
Early adolescence	36 (11.4%)	536 (14.4%)	
Late adolescence	164 (51.7%)	1643 (44.2%)	
Adulthood	117 (36.9%)	1538 (41.4%)	
Ethnic, n (%)			< 0.001
Mexican American	28 (8.8%)	786 (21.1%)	
Non-Hispanic Black	52 (16.4%)	719 (19.3%)	
Non-Hispanic White	222 (70.0%)	1897 (51.0%)	
Other Hispanic	5 (1.6%)	170 (4.6%)	
Other Race—Including Multi-Racial	10 (3.2%)	145 (3.9%)	
Annual family income, n (%)			0.105
$0 to $4,999	9 (2.8%)	158 (4.3%)	
$5,000 to $9,999	15 (4.7%)	200 (5.4%)	
$10,000 to $14,999	18 (5.7%)	278 (7.5%)	
$15,000 to $19,999	18 (5.7%)	252 (6.8%)	
$20,000 to $24,999	22 (6.9%)	301 (8.1%)	
$25,000 to $34,999	36 (11.4%)	425 (11.4%)	
$35,000 to $44,999	31 (9.8%)	357 (9.6%)	
$45,000 to $54,999	22 (6.9%)	355 (9.6%)	
$55,000 to $64,999	26 (8.2%)	247 (6.6%)	
$65,000 to $74,999	23 (7.3%)	214 (5.8%)	
$75,000 and over	92 (29.0%)	808 (21.7%)	
Over $20,000 (not specified)†	4 (1.3%)	72 (1.9%)	
Under $20,000 (not specified)†	1 (0.3%)	50 (1.3%)	
BMI, kg/m^2^ (median [IQR])	27.24 [23.36, 32.29]	27.18 [23.29, 32.59]	0.720
Education level, n (%)			< 0.001
Less than 9th Grade	5 (1.6%)	210 (5.6%)	
9-11th Grade (Includes 12th grade with no diploma)	27 (8.5%)	552 (14.9%)	
High School Grade/GED or Equivalent	89 (28.1%)	816 (22.0%)	
Some College or AA degree	114 (36.0%)	1232 (33.1%)	
College Graduate or above	82 (25.9%)	907 (24.4%)	
Smoking status‡, n (%)			
Non-smoker	154 (48.6%)	2074 (55.8%)	0.010
Former smoker	61 (19.2%)	723 (19.5%)	
Current smoker	102 (32.2%)	920 (24.8%)	
Alcohol consumption§, n (%)			0.465
Non-drinker	68 (21.5%)	694 (18.7%)	
Moderate drinker	245 (77.3%)	2968 (79.8%)	
Heavy drinker	4 (1.3%)	55 (1.5%)	
Hypertension, n (%)			0.006
Yes	76 (24.0%)	653 (17.6%)	
No	241 (76.0%)	3064 (82.4%)	
Diabetes status, n (%)			0.977
Yes	12 (3.8%)	149 (4.0%)	
Borderline	3 (0.9%)	37 (1.0%)	
No	302 (95.3%)	3531 (95.0%)	
Used DepoProvera or injectables, n (%)			0.001
Yes	30 (9.5%)	617 (16.6%)	
No	287 (90.5%)	3100 (83.4%)	

Continuous variables are presented as mean ± SD or median [IQR] as appropriate and compared using Student’s t-test or Mann–Whitney U test accordingly. Categorical variables are presented as n (%), and compared using Pearson’s chi-square test. All *P* values were two-tailed and a value of *P* < 0.05 was considered statistically significant

*Abbreviations*: *SD* standard deviation, *IQR* Interquartile range, *AFS* Age at first sexual intercourse, *BMI* Body mass index, *GED* General educational development, *AA* Associate's degree

^*^: AFS groups: 10 < AFS ≤ 15 years old as early adolescence, 15 < AFS < 18 years old as late adolescence, and 18 ≤ AFS ≤ 30 years old as adulthood. AFS is the exposure variable of interest and is displayed here to show its distribution by outcome status

^†^: Some participants (mostly from the 1999–2000 NHANES cycle) selected only “Under $20,000” or “Over $20,000” without reporting a more specific income range. These are presented as separate categories

^‡^: Non-smokers were defined as individuals who have smoked fewer than 100 cigarettes in their lifetime, former smokers are those who have smoked more than 100 cigarettes but are not currently smoking, and current smokers are those who currently smoke regularly

^§^: Non-drinkers were defined as those consuming 0 drinks per year, moderate drinkers as those consuming less than 1 drink per day, and heavy drinkers as those consuming 1 or more drinks per day

### Population characteristics of UK Biobank

This study included 174,933 women from the UK Biobank cohort, of whom 4,155 were diagnosed with endometriosis and 170,778 served as controls. Participants were categorized into three groups based on their age at first sexual intercourse (AFS): early adolescence (10 < AFS ≤ 15 years, 4.5% in the endometriosis group vs. 3.0% in controls), late adolescence (15 < AFS < 18 years, 43.3% vs. 33.8%), and adulthood (18 ≤ AFS ≤ 30 years, 52.2% vs. 63.2%) (Table 2). There was no significant difference between endometriosis group and control group regarding the prevalence of type 2 diabetes mellitus (*P* = 0.833) or hypertension (*P* = 0.06). Significant differences between groups were observed in ethnicity, income level, Townsend deprivation index, BMI, education level, smoking status, and alcohol consumption (all *P* < 0.05) (Table 2).

**Table 2:** Baseline characteristics of included patients from UK Biobank

	Endometriosis (*N* = 4,155)	Controls (*N* = 170,778)	*P* value
AFS group^*^, n (%)			< 0.001
Early adolescence	188 (4.5%)	5,129 (3.0%)	
Late adolescence	1,798 (43.3%)	57,759 (33.8%)	
Adulthood	2,169 (52.2%)	107,890 (63.2%)	
Ethnic†, n (%)			< 0.001
White	3,945 (94.9%)	164,085 (96.1%)	
Black	83 (2.0%)	2,331 (1.4%)	
Asian	49 (1.2%)	1,692 (1.0%)	
Mixed	44 (1.1%)	1,111 (0.7%)	
Chinese	4 (0.1%)	402 (0.2%)	
Other	30 (0.7%)	1,157 (0.7%)	
Annual household income, n (%)			< 0.001
Less than £18,000	833 (20.0%)	40,992 (24.0%)	
£18,000 to £30,999	1,033 (24.9%)	45,335 (26.5%)	
£31,000 to £51,999	1,180 (28.4%)	44,101 (25.8%)	
£52,000 to £100,000	960 (23.1%)	32,297 (18.9%)	
Greater than £100,000	149 (3.6%)	8,053 (4.7%)	
Townsend deprivation index‡ (median [IQR])	−1.89 [−3.48, 0.73]	−2.17 [−3.65, 0.37]	< 0.001
BMI, kg/m^2^ (median [IQR])	26.73 [23.81, 30.60]	26.10 [23.46, 29.73]	< 0.001
Education level§, n (%)			< 0.001
No education	402 (9.7%)	23,314 (13.7%)	
Vocational education	728 (17.5%)	26,778 (15.7%)	
Secondary education	1,685 (40.6%)	62,479 (36.6%)	
Higher education	1,340 (32.3%)	58,207 (34.1%)	
Smoking status, n (%)			
Current	450 (10.8%)	16,165 (9.5%)	< 0.001
Never	2,492 (60.0%)	97,975 (57.4%)	
Previous	1,213 (29.2%)	56,638 (33.2%)	
Alcohol consumption, n (%)			0.02
Current	3,829 (92.2%)	157,390 (92.2%)	
Never	156 (3.8%)	7,451 (4.4%)	
Previous	170 (4.1%)	5,937 (3.5%)	
Hypertension, n (%)			0.06
Yes	1,152 (27.7%)	49,663 (29.1%)	
No	3,003 (72.3%)	121,115 (70.9%)	
Type 2 diabetes mellitus, n (%)			0.833
Yes	261 (6.3%)	10,887 (6.4%)	
No	3,894 (93.7%)	159,891 (93.6%)	

Continuous variables are presented as mean ± SD or median [IQR] as appropriate and compared using Student’s t-test or Mann–Whitney U test accordingly. Categorical variables are presented as n (%), and compared using Pearson’s chi-square test. All *P* values were two-tailed and a value of *P* < 0.05 was considered statistically significant

*Abbreviations*: *SD* standard deviation, *IQR* Interquartile range, *AFS* Age at first sexual intercourse, *BMI* Body mass index

^*^: AFS groups: 10 < AFS ≤ 15 years old as early adolescence, 15 < AFS < 18 years old as late adolescence, and 18 ≤ AFS ≤ 30 years old as adulthood. AFS is the exposure variable of interest and is displayed here to show its distribution by outcome status

^†^: Ethnicity was self-reported and categorized according to UK Biobank standards: White, Black, Asian (including Indian, Pakistani, and Bangladeshi), Chinese, Mixed, and Other

^‡^: The Townsend deprivation index (TDI) was calculated at baseline based on participants’ postcode of residence, using census data on unemployment, car ownership, home ownership, and household overcrowding. A higher score indicates greater socioeconomic deprivation

^§^: Educational attainment was self-reported at baseline and categorized as follows: no education; vocational education (e.g., National Vocational Qualification [NVQ], Higher National Certificate [HNC], Higher National Diploma [HND], or other professional certifications); secondary education (e.g., General Certificate of Secondary Education [GCSEs], Certificate of Secondary Education [CSEs], Ordinary Level [O-levels], and Advanced Level [A-levels]); and higher education (college or university degree)

### Association between AFS and endometriosis

In the NHANES cohort, although the early adolescence group did not show a significant association in either analysis, the robust association for the late adolescence group persisted even after adjusting for multiple confounders. Either in the unadjusted model (model 1) or adjusted model (model 2), women who initiated sexual intercourse during late adolescence had a significantly higher risk of endometriosis compared with those who began in adulthood. The LRT test for NHANES data yielded a chi-squared (*χ*^*2*^) value of 55.19 with 21 degrees of freedom (*P* = 6.63 × 10^–5^), indicating that model 2 provides a significantly improved fit over the unadjusted model. Women who had their first sexual intercourse at late adolescence had a 31% higher risk of endometriosis (Odds ratio [OR] = 1.31, 95% confidence interval [CI]: 1.01–1.70, *P* = 0.04) compared to those who initiated sexual activity in adulthood.

In the UK Biobank cohort, the association between earlier AFS and endometriosis risk was even more pronounced. In the unadjusted model (model 1), early adolescent sexual initiation was associated with an 82% increased risk of endometriosis (OR = 1.82, 95% CI: 1.57–2.12, *P* < 0.001), and late adolescence with a 55% increase (OR = 1.55, 95% CI: 1.45–1.65, *P* < 0.001), compared to adulthood. These associations remained significant in the adjusted model (model 2), where the odds ratios were slightly attenuated but still substantial: OR = 1.73 (95% CI: 1.48–2.02, *P* < 0.001) for early adolescence and OR = 1.51 (95% CI: 1.41–1.61, *P* < 0.001) for late adolescence. The consistent findings across both models and cohorts suggest that initiating sexual activity before adulthood is associated with an increased risk of endometriosis (Table 3).

**Table 3:** Logistic regression analysis of the association between AFS and risk of endometriosis

AFS^*^	*β*	OR (95% CI)	*P*-value		*β*	OR (95% CI)	*P*-value
US NHANES				UK Biobank			
Model 1 (unadjusted)†				Model 1 (unadjusted)†			
Adulthood		1.00 (Reference)		Adulthood		1.00 (Reference)	
Early adolescence	− 0.12	0.88 (0.60–1.30)	0.53	Early adolescence	0.60	1.82 (1.57–2.12)	< 0.001
Late adolescence	0.27	1.31 (1.03–1.68)	0.03	Late adolescence	0.44	1.55 (1.45–1.65)	< 0.001
Model 2 (adjusted)‡				Model 2 (adjusted)§			
Adulthood		1.00 (Reference)		Adulthood		1.00 (Reference)	
Early adolescence	− 0.08	0.92 (0.61–1.37)	0.67	Early adolescence	0.55	1.73 (1.48–2.02)	< 0.001
Late adolescence	0.27	1.31 (1.01–1.70)	0.04	Late adolescence	0.41	1.51 (1.41–1.61)	< 0.001
	*Δdf*¶	*χ*^*2*^#	*P*-value				
LRT	21	55.19	6.63E-05				

*Abbreviations*: *AFS* Age at first sexual intercourse, *OR* Odds ratio, *CI* Confidence interval, *LRT* Likelihood ratio test, *BMI* Body mass index

^*^: AFS groups: 10 < AFS ≤ 15 years old as early adolescence, 15 < AFS < 18 years old as late adolescence, and 18 ≤ AFS ≤ 30 years old as adulthood

^†^: unadjusted model

^‡^: adjusted for ethnic, annual family income, BMI, smoking status, alcohol consumption, hypertension, diabetes and used Depo-Provera or injectables

^§^: adjusted for ethnic, education, income, BMI, smoking status, alcohol consumption, Townsend deprivation index at recruitment, hypertension, type 2 diabetes mellitus

^¶^: change in degrees of freedom

^#^: chi-square statistic

### MR of AFS and endometriosis

**Fig. 2:**
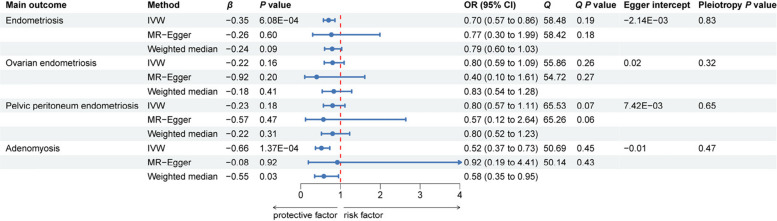
Forest plot for the MR analysis of the association between AFS and endometriosis and adenomyosis. Abbreviations: AFS: Age at first sexual intercourse; IVW: Inverse-variance weighted; OR: Odds ratios; CI: Confidence interval. All *P* values were two-tailed and a value of *P* < 0.05 was considered statistically significant

The use of MR analysis helps mitigate confounding and reverse causation, providing robust evidence for the causal relationship between AFS and the risk of endometriosis and adenomyosis. The IVW method demonstrated that delayed AFS was associated with a significantly lower risk of endometriosis and adenomyosis. Specifically, for endometriosis, the IVW analysis yielded a *β* coefficient of − 0.35, corresponding to an OR of 0.70 (95% CI: 0.57–0.86, *P* = 6.08 × 10^–4^), indicating a 30% reduced risk of developing endometriosis per year delay in AFS. For adenomyosis, the IVW method produced a *β* of − 0.66 (OR = 0.52, 95% CI: 0.37–0.73, *P* = 1.37 × 10^–4^), suggesting a 48% lower risk of developing adenomyosis per year delay in AFS. Furthermore, the IVW estimates showed a *β* of − 0.22 (OR = 0.80, 95% CI: 0.59–1.09, *P* = 0.16) for ovarian endometriosis and a *β* of − 0.23 (OR = 0.80, 95% CI: 0.57–1.11, *P* = 0.18) for peritoneal endometriosis. Although the associations between delayed AFS and the risk of ovarian and peritoneal endometriosis didn’t reach statistical significance, they exhibited a similar protective trend (Fig. 2). Scatter plots displaying the results of the univariable analysis can be found in Supplementary Fig. 1.

### Horizontal pleiotropy assessment and sensitivity analysis

To ensure the robustness of the findings, several sensitivity analyses were conducted. The MR-Egger intercept test and MR-PRESSO global test were used to assess for horizontal pleiotropy. The results indicated no evidence of horizontal pleiotropy or heterogeneity, confirming the validity of the genetic instruments used in the analysis (Fig. 2). Additionally, funnel plots for heterogeneity testing are included in Supplementary Fig. 2, while Supplementary Fig. 3 presents the outcomes of the leave-one-SNP-out analysis. These findings support the reliability of the causal estimates obtained and underscore the importance of considering genetic pleiotropy in MR studies to avoid biased results.

## Discussion

### Principal findings

Our findings highlight the potential impact of early sexual behavior on female reproductive health, particularly the risk of endometriosis. The IVW MR analysis further reinforces the possibility of a causal relationship between AFS and the risk of developing endometriosis.

### Results in the context of what is known

Our findings align with and expand upon existing literature concerning the impact of AFS on gynecological health, particularly endometriosis and adenomyosis. Previous studies have reported associations between early sexual initiation and adverse reproductive health outcomes, including hormonal imbalances, increased risks of pelvic infections, and inflammatory responses that contribute to the pathogenesis of endometriosis [28, 37–40]. In the NHANES cohort, late adolescent AFS was associated with an approximately 31% higher risk of endometriosis compared with adulthood, indicating a consistent direction of effect despite differences in cohort design and outcome ascertainment. Importantly, the concordance of effect direction across cohorts supports the robustness of the observed association rather than cohort-specific bias.

In the UK Biobank cohort of more than 170,000 women, earlier AFS was consistently associated with an increased risk of endometriosis across models using hospital-record–based diagnoses. Compared with initiation in adulthood, early adolescent AFS was associated with a 73% higher risk (OR = 1.73, 95% CI: 1.48–2.02), and late adolescent AFS with a 51% higher risk (OR = 1.51, 95% CI: 1.41–1.61). The narrow confidence intervals reflect the large sample size and provide precise population-level estimates that complement the NHANES findings. The overall pattern of associations remains consistent with earlier observational studies linking early sexual initiation to increased susceptibility to reproductive disorders [23, 26, 41, 42].

The IVW MR analysis further strengthens the evidence by demonstrating a potential causal relationship, showing that genetically predicted delayed AFS was significantly associated with an about 30% lower risk of endometriosis and an about 48% lower risk of adenomyosis. These results are robust, with no evidence of horizontal pleiotropy or heterogeneity affecting the findings, and they suggest that interventions aimed at delaying sexual initiation could have a protective effect against these conditions.

### Clinical implications

The findings of this study highlight the potential importance of early-life behavioral and psychosocial factors in shaping long-term reproductive health. Previous studies have also suggested that early sexual activity may lead to hormonal imbalances, chronic inflammation, and alterations in the vaginal microbiome, which could contribute to the pathogenesis of endometriosis [28, 37–40]. AFS is a complex trait shaped by family environment, education, social context, and mental health, and therefore should not be interpreted as a simple or isolated modifiable exposure. While the MR analysis provides evidence supporting a causal association between AFS and the risks of overall endometriosis and adenomyosis, this relationship does not appear to be uniform across all endometriosis subtypes.

Rather than viewing AFS as a direct and universal causal factor, our findings suggest that early sexual initiation may reflect broader behavioral and psychosocial vulnerability that contributes to disease risk in specific gynecological phenotypes. From a public health perspective, these results highlight the importance of comprehensive sexual health education, psychosocial support for adolescents, and early identification of individuals at higher risk. Adolescents and their guardians should be informed about the potential long-term reproductive health risks associated with early sexual activity. Conversely, delaying AFS may reduce the exposure to sexually transmitted infections (STIs), help maintain hormonal balance during critical periods of reproductive development, and lower the risk of adverse gynecological outcomes [43]. Our findings underscore the potential importance of public health policies and educational programs aimed at delaying adolescent sexual activity. Such initiatives can help mitigate the risks associated with early sexual initiation and promote better reproductive health outcomes for women [44].

Moreover, early detection strategies should be emphasized in women with a history of early sexual activity, as they may be at higher risk for these conditions [21]. Regular gynecological exams and prompt investigation of symptoms such as chronic pelvic pain and abnormal menstruation can facilitate early diagnosis and more effective management of endometriosis and adenomyosis.

### Research implications

Early initiation of sexual activity, encompassing history of sexual violence and abuse and unprotected sexual activity has been associated with increased risks of STIs and unintended pregnancies [45, 46]. Firstly, multiple sexual partners or unprotected sexual contact, along with the risk of contracting high-risk types of human papillomavirus (HPV) and STIs, can cause chronic pelvic inflammation and subsequent development of endometriosis [47, 48]. Additionally, unintended pregnancies during adolescence often result in fetal demise and repeated abortions, particularly in younger women, have been suggested as potential risk factors for the development and exacerbation of endometriosis [49]. This trauma can trigger inflammatory responses, creating an environment conducive to the implantation and growth of ectopic endometrial tissue. Furthermore, sexual violence can negatively impact the mental health of adolescents, leading to anxiety and depression, which are also psychiatric comorbidities associated with endometriosis [50]. A recent large-scale population-based cohort study conducted in Sweden found that adverse childhood experiences, particularly exposure to violence, were significantly associated with an increased risk of developing endometriosis later in life, highlighting the role of early environmental factors in shaping long-term reproductive health vulnerabilities [51]. Chronic stress can affect immune function and hormonal balance, potentially exacerbating conditions like endometriosis. Hormonal dysregulation can lead to irregular menstrual cycles and altered endometrial shedding, which may facilitate the retrograde menstruation theory of endometriosis [52]. The risk of endometriosis appears to be reduced with the use of oral contraceptives, which significantly decrease menstrual flow and may hypothetically interfere with the implantation of refluxed endometrial cells [53].

The relationship established between AFS and the risks of endometriosis and adenomyosis opens new avenues for research. Factors such as education level, access to sexual health resources, and cultural attitudes towards sexual behavior may influence both the timing of sexual initiation and the risk of developing endometriosis and adenomyosis. Future research should aim to disentangle these complex interactions and identify modifiable risk factors that can be targeted through public health interventions. Future studies should explore the underlying biological mechanisms linking early sexual activity to these conditions, including hormonal, inflammatory, and microbiome-related pathways.

### Strengths and limitations

This study has several notable strengths. Firstly, the study's use of multivariable logistic regression analysis stands out as a primary strength, allowing for the control of multiple confounding factors and thereby strengthening the validity of the findings. This approach ensures that the observed associations are more likely to reflect true relationships than spurious correlations. Furthermore, the study employs MR using the IVW method, which enhances causal inference by minimizing confounding and reverse causation. MR leverages genetic variants as IVs, mimicking the conditions of a randomized controlled trial, and providing robust evidence for the causal relationship between AFS and the risk of endometriosis. Additionally, the study utilizes data from the NHANES, spanning multiple cycles and including a large, representative sample of the U.S. population. This comprehensive dataset enhances the external validity and generalizability of the findings to a broader population. Furthermore, combining MR results with NHANES phenotypic analyses enriches the study by bridging causal inference with population-based observational data, providing complementary evidence and facilitating the exploration of population-level impacts. Lastly, the study conducts detailed subgroup analyses, examining the impact of AFS on different types of endometriosis and adenomyosis. This level of detail allows for a nuanced understanding of how early sexual initiation affects various gynecological conditions, offering insights that can inform targeted prevention and intervention strategies.

Several limitations should also be acknowledged. One of the limitations of the study is the reliance on self-reported data in NHANES for determining AFS and diagnosis of endometriosis and adenomyosis. Self-reported data are subject to recall bias and misreporting, which could affect the accuracy of the findings. Additionally, the cross-sectional nature of the NHANES data limits the ability to establish temporal relationships between early sexual initiation and the development of endometriosis and adenomyosis, highlighting the need for longitudinal studies to confirm these findings and better understand the temporal dynamics involved. Although we adjusted for several confounders, there may still be unmeasured variables such as childhood trauma, socioeconomic status, and access to healthcare that could influence the results.

In the UK Biobank, endometriosis cases were primarily identified through linked hospital records, which may undercapture milder cases managed in primary care or not formally diagnosed. Participants were also recruited at mid to later adulthood, which may affect recall of reproductive history. Differences in outcome ascertainment and cohort composition between NHANES and the UK Biobank may contribute to variation in effect magnitude; however, the direction of associations was consistent across cohorts, supporting the robustness of the overall findings.

The genetic variants identified in GWAS of European populations may have different allele frequencies or effect sizes in other populations, such as those represented in NHANES. This could reduce the validity and strength of the genetic instruments when applied to non-European groups, potentially impacting the interpretation of the results. While the NHANES dataset is representative of the U.S. population, the findings may not be fully generalizable to other populations with different genetic backgrounds, cultural practices, and healthcare systems, necessitating further studies in diverse populations to validate the findings and explore potential variations. Lastly, despite the use of MR to address confounding, there is always the possibility of genetic pleiotropy, where a single genetic variant influences multiple traits. While the study found no evidence of horizontal pleiotropy, it remains a potential limitation that could influence the interpretation of the results.

## Conclusion

Our study highlights the significant impact of early sexual initiation on the risk of developing endometriosis and adenomyosis. By promoting delayed sexual initiation and providing comprehensive sexual health education, one may improve overall reproductive health outcomes for women. Future research should focus on elucidating the biological mechanisms, validating findings through longitudinal studies, and exploring the influence of socioeconomic and environmental factors.

## Supplementary Information

Supplementary Material 1: Figure S1. The scatter plot of SNPs associated with AFS and endometriosis. A. Endometriosis. B. Ovarian Endometriosis. C. Pelvic Peritoneum Endometriosis. D. Adenomyosis. SNPs: Single nucleotide polymorphisms; AFS: Age at first sexual intercourse.

Supplementary Material 2: Figure S2. The funnel plot of SNPs associated with AFS and endometriosis. A. Endometriosis. B. Ovarian Endometriosis. C. Pelvic Peritoneum Endometriosis. D. Adenomyosis. SNPs: Single nucleotide polymorphisms; AFS: Age at first sexual intercourse.

Supplementary Material 3: Figure S3. The leave-one-out test plot of SNPs associated with AFS and endometriosis. A. Endometriosis. B. Ovarian Endometriosis. C. Pelvic Peritoneum Endometriosis. D. Adenomyosis. SNPs: Single nucleotide polymorphisms; AFS: Age at first sexual intercourse.

Supplementary Material 4: Table S1. The detailed information of AFS as IVs in the study. AFS: Age at first sexual intercourse; IVs: Instrumental variables.

Supplementary Material 5: Table S2. The basic information on the GWAS of exposure and outcome. GWAS: genome-wide association studies.

## Data Availability

The data that support the findings of this study are available in NHANES database at [https://www.cdc.gov/nchs/nhanes](https://www.cdc.gov/nchs/nhanes) and UK Biobank (application ID: 548384) at https://www.ukbiobank.ac.uk/. The data of AFS are available in IEU OpenGWAS project at https://gwas.mrcieu.ac.uk/, reference number: ebi-a-GCST90000045. The data of endometriosis are available in FinnGen at [https://www.finngen.fi](https://www.finngen.fi) . The analysis codes are available from the corresponding author on reasonable request.

## Abbreviations

AA: Associate's degree
AFS: Age at first sexual intercourse
BMI: Body mass index
CI: Confidence interval
ERβ: Estrogen receptor β
GED: General educational development
GWAS: Genome-wide association studies
HPG: Hypothalamic-pituitary–gonadal
HPV: Human papillomavirus
ICD-10: International Classification of Diseases, 10th Revision
IQR: Interquartile range
IVs: Instrumental variables
IVW: Inverse-variance-weighted
LD: Linkage disequilibrium
LRT: Likelihood ratio test
MEC: Mobile examination center
MR: Mendelian randomization
MRI: Magnetic resonance imaging
NHANES: National Health and Nutrition Examination Survey
OR: Odds ratio
SD: Standard deviation
SNP: Single nucleotide polymorphisms
STIs: Sexually transmitted infections
