# Supplementary figures and images for: Unraveling the link between early sexual initiation and endometriosis: evidence from population-based analyses and genetic causal inference
Source: Reprod Biol Endocrinol. 2026 Mar 3;24:41. doi: 10.1186/s12958-026-01539-8 (PMC13063573; doi:10.1186/s12958-026-01539-8)

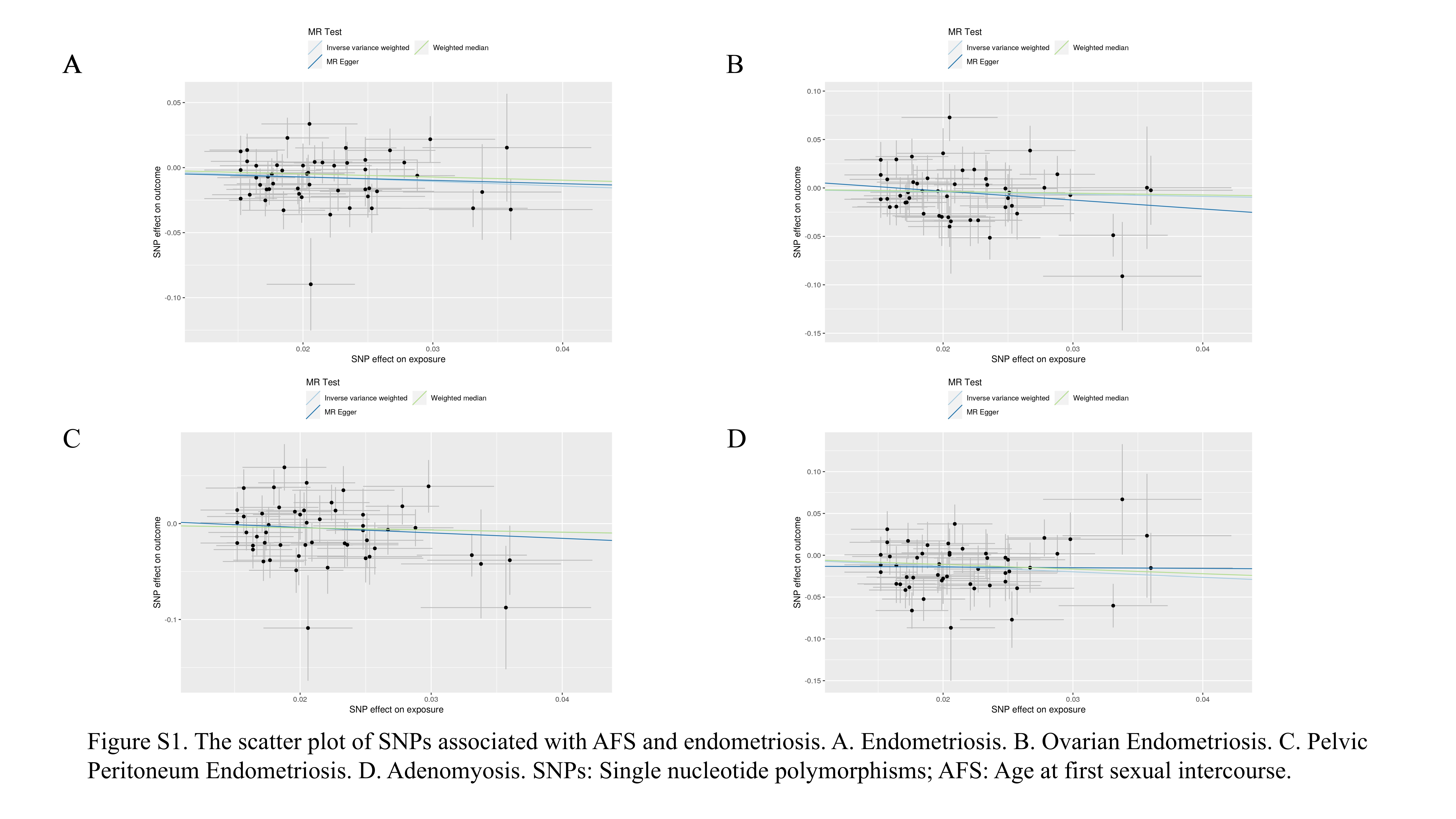

Supplement: Supplementary file 1 — Supplementary Material 1: Figure S1. The scatter plot of SNPs associated with AFS and endometriosis. A. Endometriosis. B. Ovarian Endometriosis. C. Pelvic Peritoneum Endometriosis. D. Adenomyosis. SNPs: Single nucleotide polymorphisms; AFS: Age at first sexual intercourse. [file 12958_2026_1539_MOESM1_ESM.jpg]

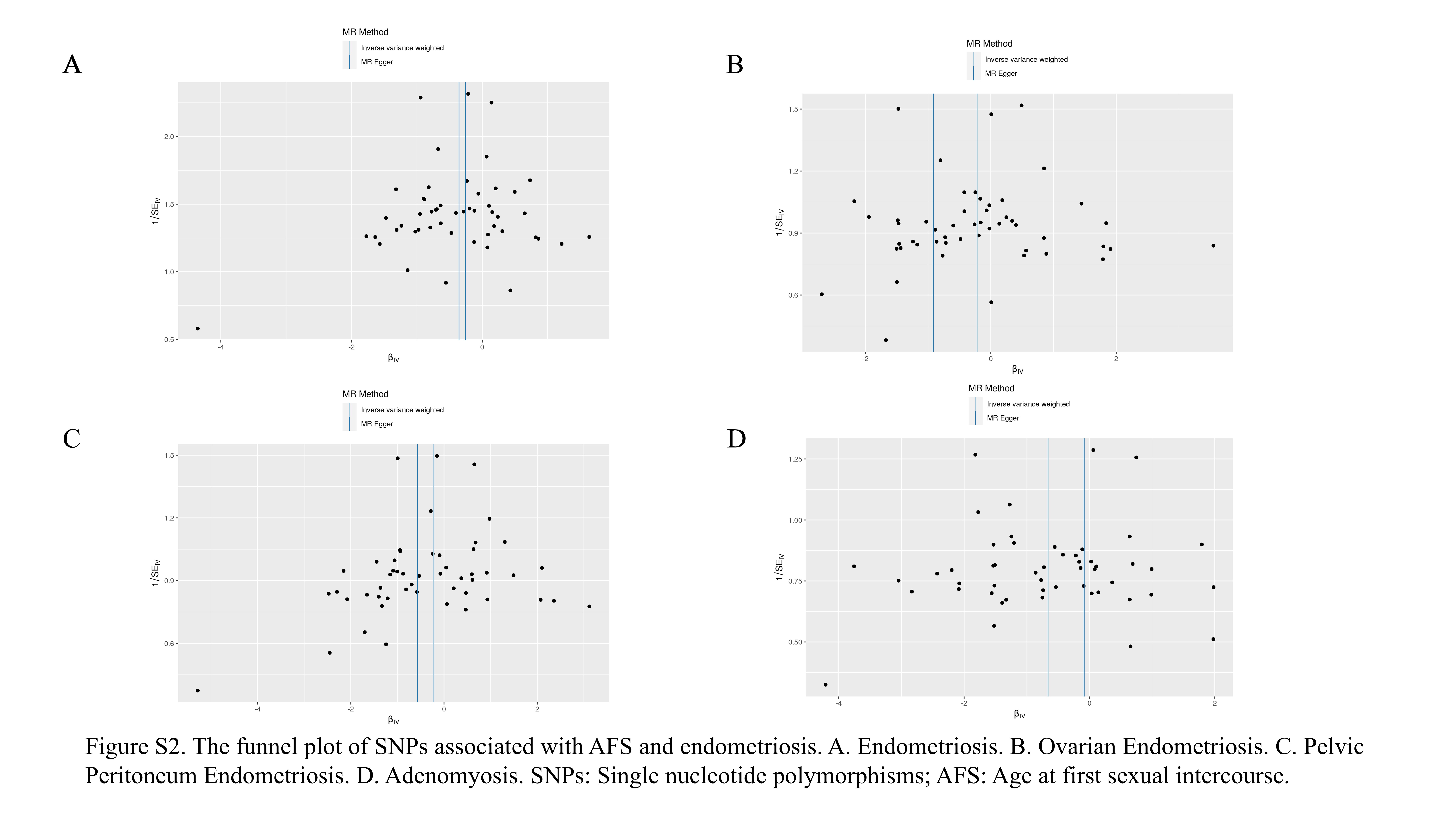

Supplement: Supplementary file 2 — Supplementary Material 2: Figure S2. The funnel plot of SNPs associated with AFS and endometriosis. A. Endometriosis. B. Ovarian Endometriosis. C. Pelvic Peritoneum Endometriosis. D. Adenomyosis. SNPs: Single nucleotide polymorphisms; AFS: Age at first sexual intercourse. [file 12958_2026_1539_MOESM2_ESM.jpg]

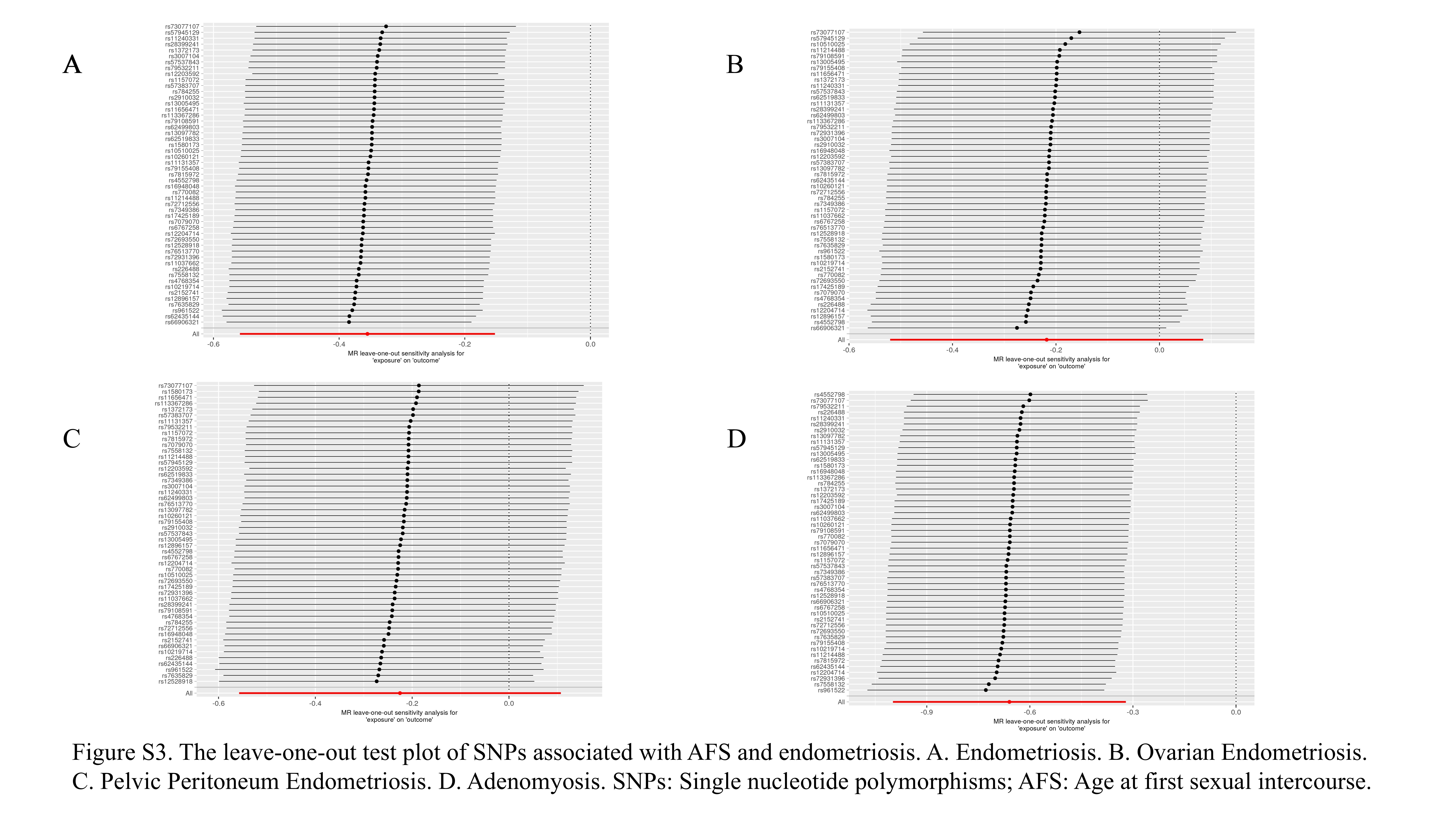

Supplement: Supplementary file 3 — Supplementary Material 3: Figure S3. The leave-one-out test plot of SNPs associated with AFS and endometriosis. A. Endometriosis. B. Ovarian Endometriosis. C. Pelvic Peritoneum Endometriosis. D. Adenomyosis. SNPs: Single nucleotide polymorphisms; AFS: Age at first sexual intercourse. [file 12958_2026_1539_MOESM3_ESM.jpg]
