# Supplementary material for: Unraveling the link between early sexual initiation and endometriosis: evidence from population-based analyses and genetic causal inference
Source: Reprod Biol Endocrinol. 2026 Mar 3;24:41. doi: 10.1186/s12958-026-01539-8 (PMC13063573; doi:10.1186/s12958-026-01539-8)
Supplement: Supplementary file 4 — Supplementary Material 4: Table S1. The detailed information of AFS as IVs in the study. AFS: Age at first sexual intercourse; IVs: Instrumental variables. [file 12958_2026_1539_MOESM4_ESM.docx]

Table S1. The detailed information of AFS as IVs in the study.

| ID | SNP | Effect allele | Other allele | *β* | SE | *P* value | EAF |
| --- | --- | --- | --- | --- | --- | --- | --- |
| ebi-a-GCST90000045 | rs17425189 | C | T | 0.02 | 0.0036 | 2.77E-08 | NA |
| ebi-a-GCST90000045 | rs1157072 | G | A | 0.036 | 0.0063 | 1.10E-08 | NA |
| ebi-a-GCST90000045 | rs11240331 | T | C | 0.0185 | 0.0032 | 7.41E-09 | NA |
| ebi-a-GCST90000045 | rs1226414 | T | A | 0.0179 | 0.0028 | 1.63E-10 | NA |
| ebi-a-GCST90000045 | rs72931396 | A | C | 0.0157 | 0.0028 | 2.06E-08 | NA |
| ebi-a-GCST90000045 | rs7349386 | C | T | 0.0357 | 0.0065 | 3.97E-08 | NA |
| ebi-a-GCST90000045 | rs57537843 | A | G | -0.0159 | 0.0029 | 4.19E-08 | NA |
| ebi-a-GCST90000045 | rs66906321 | C | T | -0.0205 | 0.0037 | 3.02E-08 | NA |
| ebi-a-GCST90000045 | rs13005495 | G | T | -0.0248 | 0.0028 | 8.21E-19 | NA |
| ebi-a-GCST90000045 | rs961522 | C | T | 0.0278 | 0.0028 | 3.13E-23 | NA |
| ebi-a-GCST90000045 | rs359240 | A | G | -0.0205 | 0.0028 | 2.45E-13 | NA |
| ebi-a-GCST90000045 | rs62166484 | A | T | -0.031 | 0.0055 | 1.74E-08 | NA |
| ebi-a-GCST90000045 | rs7558132 | A | G | -0.0209 | 0.0029 | 5.72E-13 | NA |
| ebi-a-GCST90000045 | rs13097782 | T | C | 0.0167 | 0.0028 | 2.46E-09 | NA |
| ebi-a-GCST90000045 | rs186723454 | G | A | -0.0222 | 0.0039 | 1.25E-08 | NA |
| ebi-a-GCST90000045 | rs6767258 | A | G | -0.0248 | 0.0038 | 6.74E-11 | NA |
| ebi-a-GCST90000045 | rs57945129 | T | C | 0.0236 | 0.0039 | 1.44E-09 | NA |
| ebi-a-GCST90000045 | rs7635829 | A | G | -0.0188 | 0.0032 | 4.23E-09 | NA |
| ebi-a-GCST90000045 | rs73077107 | A | G | 0.0331 | 0.0042 | 3.25E-15 | NA |
| ebi-a-GCST90000045 | rs1580173 | A | G | -0.0177 | 0.0028 | 2.59E-10 | NA |
| ebi-a-GCST90000045 | rs72712556 | A | G | 0.0184 | 0.003 | 8.61E-10 | NA |
| ebi-a-GCST90000045 | rs11131357 | T | C | 0.0164 | 0.003 | 4.59E-08 | NA |
| ebi-a-GCST90000045 | rs79532211 | G | A | 0.0253 | 0.004 | 2.53E-10 | NA |
| ebi-a-GCST90000045 | rs226488 | C | T | -0.0224 | 0.0029 | 1.13E-14 | NA |
| ebi-a-GCST90000045 | rs2910032 | T | C | 0.0174 | 0.0028 | 5.16E-10 | NA |
| ebi-a-GCST90000045 | rs12203592 | T | C | 0.0206 | 0.0034 | 1.37E-09 | NA |
| ebi-a-GCST90000045 | rs12204714 | T | C | 0.0288 | 0.0029 | 3.05E-23 | NA |
| ebi-a-GCST90000045 | rs12528918 | G | T | -0.018 | 0.0028 | 1.29E-10 | NA |
| ebi-a-GCST90000045 | rs4552798 | T | C | 0.0176 | 0.0028 | 3.26E-10 | NA |
| ebi-a-GCST90000045 | rs62435144 | A | G | 0.0298 | 0.005 | 2.52E-09 | NA |
| ebi-a-GCST90000045 | rs10260121 | C | T | -0.0251 | 0.0043 | 5.31E-09 | NA |
| ebi-a-GCST90000045 | rs113367286 | T | C | 0.0172 | 0.0031 | 2.88E-08 | NA |
| ebi-a-GCST90000045 | rs198310 | T | A | 0.02 | 0.0034 | 4.04E-09 | NA |
| ebi-a-GCST90000045 | rs12541633 | T | A | 0.0156 | 0.0028 | 2.53E-08 | NA |
| ebi-a-GCST90000045 | rs28399241 | T | C | -0.0171 | 0.0028 | 1.01E-09 | NA |
| ebi-a-GCST90000045 | rs62499803 | G | A | -0.0199 | 0.0036 | 3.24E-08 | NA |
| ebi-a-GCST90000045 | rs7815972 | G | A | 0.0173 | 0.0029 | 2.44E-09 | NA |
| ebi-a-GCST90000045 | rs62519833 | G | A | 0.0257 | 0.0044 | 5.19E-09 | NA |
| ebi-a-GCST90000045 | rs1372173 | G | A | 0.0221 | 0.0037 | 2.33E-09 | NA |
| ebi-a-GCST90000045 | rs72693550 | A | C | -0.0215 | 0.0038 | 1.53E-08 | NA |
| ebi-a-GCST90000045 | rs10761784 | T | A | 0.0152 | 0.0028 | 5.68E-08 | NA |
| ebi-a-GCST90000045 | rs10510025 | T | C | -0.0205 | 0.0032 | 1.49E-10 | NA |
| ebi-a-GCST90000045 | rs7079070 | A | G | -0.0164 | 0.0028 | 4.71E-09 | NA |
| ebi-a-GCST90000045 | rs11037662 | T | G | 0.0248 | 0.004 | 5.65E-10 | NA |
| ebi-a-GCST90000045 | rs7942078 | T | A | 0.0178 | 0.0029 | 8.36E-10 | NA |
| ebi-a-GCST90000045 | rs11214488 | T | C | -0.0204 | 0.0036 | 1.46E-08 | NA |
| ebi-a-GCST90000045 | rs10219714 | T | C | 0.0157 | 0.0029 | 6.17E-08 | NA |
| ebi-a-GCST90000045 | rs770082 | A | G | -0.0152 | 0.0028 | 5.68E-08 | NA |
| ebi-a-GCST90000045 | rs4768354 | T | C | 0.0152 | 0.0028 | 5.68E-08 | NA |
| ebi-a-GCST90000045 | rs79155408 | T | C | -0.0338 | 0.0061 | 3.01E-08 | NA |
| ebi-a-GCST90000045 | rs2152741 | T | C | 0.0233 | 0.0039 | 2.31E-09 | NA |
| ebi-a-GCST90000045 | rs3007104 | A | G | -0.0152 | 0.0028 | 5.68E-08 | NA |
| ebi-a-GCST90000045 | rs12896157 | A | G | -0.0267 | 0.0035 | 2.37E-14 | NA |
| ebi-a-GCST90000045 | rs11627661 | C | G | -0.017 | 0.0029 | 4.57E-09 | NA |
| ebi-a-GCST90000045 | rs79108591 | A | G | -0.0227 | 0.0034 | 2.45E-11 | NA |
| ebi-a-GCST90000045 | rs76513770 | C | T | 0.0234 | 0.0041 | 1.15E-08 | NA |
| ebi-a-GCST90000045 | rs16948048 | G | A | -0.0203 | 0.0029 | 2.56E-12 | NA |
| ebi-a-GCST90000045 | rs11656471 | T | G | -0.0197 | 0.0036 | 4.44E-08 | NA |
| ebi-a-GCST90000045 | rs140504125 | T | C | -0.0197 | 0.0031 | 2.09E-10 | NA |
| ebi-a-GCST90000045 | rs57383707 | T | C | -0.025 | 0.0044 | 1.33E-08 | NA |
| ebi-a-GCST90000045 | rs784255 | T | G | -0.0196 | 0.0028 | 2.56E-12 | NA |

Abbreviations: AFS: Age at first sexual intercourse; IVs: Instrumental variables; SNP: Single nucleotide polymorphism; SE: Standard error; EAF: Effect allele frequency; NA: Not applicable.
