# Supplementary material for: Unraveling the link between early sexual initiation and endometriosis: evidence from population-based analyses and genetic causal inference
Source: Reprod Biol Endocrinol. 2026 Mar 3;24:41. doi: 10.1186/s12958-026-01539-8 (PMC13063573; doi:10.1186/s12958-026-01539-8)
Supplement: Supplementary file 5 — Supplementary Material 5: Table S2. The basic information on the GWAS of exposure and outcome. GWAS: genome-wide association studies. [file 12958_2026_1539_MOESM5_ESM.docx]

Table S2. The basic information on the GWAS of exposure and outcome.

| GWAS Source | Author | Year | Trait | Number of sample Size | Number of cases and Controls | GWAS ID | Consortium | Population | Number of SNPs |
| --- | --- | --- | --- | --- | --- | --- | --- | --- | --- |
| PMID: 34211149 | Mills MC | 2021 | Age at first sexual intercourse | 214,547 | NA | ebi-a-GCST90000045 | UK biobank | European | 16,426,473 |
| NA | NA | 2023 | Endometriosis | 128,171 | 16,588 cases and 111,583 controls | NA | The FinnGen Biobank | European | 21,311,942 |
| NA | NA | 2023 | Ovarian Endometriosis | 118,027 | 6,444 cases and 111,583 controls | NA | The FinnGen Biobank | European | 21,311,942 |
| NA | NA | 2023 | Pelvic Peritoneum Endometriosis | 117,820 | 6,237 cases and 111,583 controls | NA | The FinnGen Biobank | European | 21,311,942 |
| NA | NA | 2023 | Adenomyosis | 116,248 | 4,665 cases and 111,583 controls | NA | The FinnGen Biobank | European | 21,311,942 |

Abbreviations: GWAS: Genome-wide association studies; SNP: Single nucleotide polymorphisms; NA: Not applicable.
